# Chest closure without drainage after open patent ductus arteriosus ligation in Ugandan children: A non blinded randomized controlled trial

**DOI:** 10.1186/s12893-016-0182-x

**Published:** 2016-09-29

**Authors:** Naomi Kebba, Tom Mwambu, Michael Oketcho, Jonathan Izudi, Ekwaro A. Obuku

**Affiliations:** 1 Department of Surgery, School of Medicine, College of Health Sciences, Makerere University, P.O. Box 7072, Kampala, Uganda; 2 Uganda Heart Institute, Mulago National Teaching and Referral Hospital, P.O. Box 7051, Kampala, Uganda; 3 Institute of Public Health and Management, International Health Sciences University, P. O. Box 7782, Kampala, Uganda; 4 Department of Anatomy, Uganda Society for Health Scientists, Makerere University College of Health Sciences, Kampala, Uganda; 5 Clinical Epidemiology Unit, Department of Medicine, School of Medicine, College of Health Sciences, Makerere University, P.O. Box 7072, Kampala, Uganda; 6 Department of Epidemiology and Population Health, London School of Hygiene and Tropical Medicine, University of London, Keppel St, London, WC1E 7HU UK

**Keywords:** Patent ductus arteriosus, Ligation, Chest drain

## Abstract

**Background:**

There is clinical equipoise regarding post-operative management of patients with patent ductus arteriosus (PDA) without insertion of a chest drain. This study evaluated post operative outcomes of chest closure with or without a drain following Patent Ductus Arteriosus ligation among childen at Uganda Heart Instritute (UHI).

**Methods:**

This was an open label randomized controlled trial of 62 children 12 years of age and below diagnosed with patent ductus arteriosus at Mulago National Teaching and Referral Hospital, Uganda. Participants were randomized in the ratio of 1:1 with surgical ligation of patent ductus arteriosus to either thoracotomy closure with a chest tube or without a chest tube. All participants received standard care and were monitored hourly for 24 hours then until hospital discharge. The combined primary endpoint consisted of significant pleural space accumulation of fluid or air, higher oxygen need or infection of the surgical site. Analysis was conducted by multivariable logistic regression analysis at 5 % significance level.

**Results:**

We enrolled 62 participants, 46 (74 %) of whom were females. Their median age was 12 months (IQR: 8–36). Participants in the no-drain arm significantly had less post-operative complications compared to the drain arm (Unadjusted odds ratio [uOR]: 0.21, 95 % CI: 0.06–0.73, *p* = 0.015). This “protective effect” remained without statistical significance in the multivariable regression model (Adjusted odds ratio [aOR]: 0.07, 95 % CI: 0.00–2.50, *p* = 0.144).

**Conclusion:**

Children aged below 6 years with patent ductus arterious can safely and effectively have thoracotomy closure without using a drain in uncomplicated surgical ligation of the PDA. Chest drain was associated with post-operative complications.

**Trial registration:**

The trial was registered in the Pan African Clinical Trials registry on 1st/July/2012, retrospectively registered. Identifier number PACTR201207000395469.

**Electronic supplementary material:**

The online version of this article (doi:10.1186/s12893-016-0182-x) contains supplementary material, which is available to authorized users.

## Background

Patent ductus arteriosus (PDA) is the persistence of the normal fetal connection between the pulmonary artery and the descending aorta [1]. In full-term newborns, the ductus arteriosus (DA) routinely closes within 1 to 5 days after delivery [1]. PDA is the commonest congenital cardiac lesion encountered. It accounts for 54 % of all cardiac surgical operations at UHI.

Surgical closure of PDA is done to prevent or manage complications like congestive heart failure, recurrent pneumonia, failure to thrive, pulmonary hypertension and endocarditis [2]. Usually, it is performed via a left thoracotomy, which at times requires the insertion of a chest drain after completion of the procedure to evacuate air and/or blood from the pleural space [3]. This however is associated with a pleural reaction because the chest tube acts as a foreign body resulting in fibrin collection [4]. The chest tubes with smaller diameters become obstructed early due to collection of blood clots or fibrin thus requiring replacement [3].

In addition, chest tubes are associated with increased postoperative thoracotomy pain, compromised pulmonary function resulting in poor inspiratory effort, post-operative lung collapse and low oxygen saturations [5]. Research evidence also indicates that one in every five patients suffer pneumothorax after tube removal [6–8]. Furthermore, removal of the chest drain in young children is cumbersome and requires additional skill to minimize occurrence of pneumothorax because the child is unable to follow the instructions given during chest drain removal [2].

Retrospective studies indicate no added risk of complications to patients if routine chest drainage was omitted after uncomplicated ligation of the PDA [3, 9]. Currently, there are no studies that evaluated post-operative outcomes of PDA ligation without chest drainage in low and middle-income countries.

Our study therefore evaluated the safety of chest cavity closure without a drain following uncomplicated PDA ligation among Ugandan children at Mulago Teaching and National Referral Hospital.

## Methods

### Study setting

The study was carried out at Uganda Heart Institute (UHI) Mulago National Teaching and Referral Hospital. UHI is Uganda’s only center of cardiac surgery performing PDA ligations for over 20 years.

### Study participants

Participants were recruited from the pediatric outpatient clinic at Uganda Heart Institute and came from all parts of the country.

### Inclusion criteria

Any child aged 12 years and below with a confirmed diagnosis of PDA, and whose parents had consented to the study.

### Exclusion criteria

Children with history of wheeze/bronchospasms at pre-operative assessment, other chest surgery at the time of PDA ligation, intraoperative damage to thoracic duct or ductus arteriosus noticed during surgery and severe failure to thrive were excluded.

### Sampling and data collection

Participants were recruited from the UHI by consecutive sampling method. Eligible participants were entered in the surgery logbook and reserved for surgery on different days of the week. From this group, 62 study participants were sampled for the study. We performed a full physical exam and laboratory investigations. Venous blood was drawn for complete blood count, liver and kidney function tests, HIV serology and blood booked for emergency transfusion. All patients had a preoperative chest x-ray and ECHO done. Following clinical data collection, additional data was collected using researcher-administered questionnaires on sociodemographic, preoperative, intraoperative and postoperative patient variables.

### Endpoints

In our study, the combined primary endpoint constituted of significant pleural space accumulation (pleural separation >30 mm) [10] at 48 h post-operatively or need for increased postoperative oxygen supplementation (>1 L/min) or drainage site infection (seen as yellowish discharge at the tube drain site).

The secondary endpoint was length of stay beyond 24 h in the intensive care unit (ICU) after surgery.

### Sample size estimation

The Pocock formula was used to calculate the sample size [11]. The effect estimate was obtained from a previous study [3], which showed the outcome of surgery in the drain compared to the no-drain arm to be 28.1 % versus 6.2 %. This gave a total of 86 participants (43 per arm) for the study assuming 80 % power and 5 % significance level. There was no adjustment for loss to follow up since the time to outcome was short (48 h) to expect substantial loss.

### Randomization

An independent statistician generated random sequence codes by computer. Study participants were then randomly assigned in permuted blocks of four or six to receive surgical ligation of the PDA with either post-operative chest tube or without. Since the sample size was relatively small we anticipated that without restricted randomization imbalances would be more likely. Hence using permuted blocks of four or six would ensure adequate allocation concealment whilst maintaining balance in the different characteristics. The post-operative allocation of study participants to chest tube drain or with no chest tube drain was concealed by use of sequentially numbered opaque sealed envelopes (SNOSE). The SNOSE were numbered from 1 to 86 and thereafter arranged in ascending order. Our research assistants picked the topmost envelope and issued the rest consecutively. Inside the envelopes were patient codes on small cards labeled either “drain” or “no-drain.”

After ligation of the PDA, at the time of closing the chest, the envelope for each participant was retrieved and the contents read to the surgeon by the research assistant. The surgeon then acted as per the instructions of the envelope either drain or no drain. All boxes with the patient codes were kept by the research assistant under lock and key. The principal investigator and surgeons were unaware of the contents of the envelope until it was opened.

### Blinding

This study was an open label randomized controlled trial, as the principal investigator, study participants and assessors all knew who was in the drain or no drain arm after randomisation.

### Assessment of post-operative outcomes

After surgery patients were immediately transferred to the ICU for monitoring for at least 24 h according to standard UHI protocols. All children received oxygen by nasal prongs mandatory for 24 h starting at 1 L per minute and would be increased accordingly if the saturations fell below 92 % to a level that maintained saturations above 92 %. A chest x-ray was done within 30 min of arrival in ICU and then repeated after 24 h. For patients who had a chest tube inserted, it was removed prior to discharge from ICU in the absence of significant chest tube drainage or when there was no clinical or radiological evidence of pleural space accumulation. Significant chest tube drainage included; fluid drainage of more than 4mls per hour for children above 72 months or fluid drainage of more than 2mls per hour for children 72 months and below.

After discharge from the ICU, the patients spent a minimum of 72 h on the general ward at UHI where close monitoring was continued. This included a full clinical exam and daily assessment of the wound dressings which, would only be changed if soiling was heavy. Once dressings were opened, the wound was assessed for signs of infection which for this study we used presence of a yellow discharge at the wound site.

As a safety measure an ultrasound of the chest was done for all patients 48 h after surgery to detect and/or quantify any pleural fluid. This was done to assess the pleural drainage in both arms. It was recorded as significant or insignificant pleural fluid collection with ≥30 mm being considered as significant.

### Statistical analysis

Using STATA version 11, analysis was conducted after collecting 72 % of estimated data due to the loss of equipoise and logistical reasons that drastically reduced the number of surgeries. Baseline categorical data was tabulated as frequencies and proportions. Because of the low numbers, medians and interquartile ranges were preferred appropriate measures of central tendency for continuous data.

The primary endpoints were categorized into a combined binary outcome. We used logistic regression analysis to compute the effect estimate. Our results were expressed using Odds Ratios (OR) and adjusted Odds Ratios (aOR) with corresponding 95 % confidence intervals (95 % CI) and p-values for both primary and secondary endpoints in comparison groups. We conducted adjusted analysis for potential confounders particularly age, sex, body-mass-index, length of thoracotomy incision, muscle sparing surgery and duration of surgery. We used the Chi-squared test for large cell counts (five onwards) and the Fisher’s exact test for smaller cell counts of less than five. Our level of statistical significance was set at a two-sided *p*-value of <0.029 for one interim and one final analysis (Table 1).

**Table 1:** Fixed nominal rule to maintain overall type I error = 0.05

	Total number of analyses planned (including final)				
Analysis	1	2	3	4	5
1^st^	α = **0.05**	0.029	0.022	0.018	0.016
2^nd^		**0.029**	0.022	0.018	0.016
3^rd^			**0.022**	0.018	0.016
4^th^				**0.018**	0.016
5^th^					**0.016**

Ref: Pocock SJ. Clinical Trials: A Practical Approach. Chichester: Wiley; 1984

The bolded numbers show the levels of significance that will be used at the corresponding analysis

### Interim analysis

The study considered the DAMOCLES guidelines for stopping studies and interim analyses. The study, a priori, anticipated halting of the study using the Fixed Nominal Rule by Stuart Pocock. Hence the study was to be primarily stopped for safety, with focus on the no-DRAIN arm if we detected >6.2 % adverse events (AE) or one Serious Adverse Event (SAE) or one Suspected Unexpected Serious Adverse Reaction (SUSAR) compared to the DRAIN arm. We stopped our study due to futility, as the cardiac surgeons were no longer willing to randomize the patients hence equipoise no longer existed.

### Ethical approval and trial protocol registration

This study was approved by the School of Medicine Research and Ethics Committee (SoMREC) of Makerere University College of Health Sciences with study number # REC REF 2012–115. We registered this protocol in the Pan African Clinical Trials registry on 1st/July/2012 with identifier number PACTR20120700039546. This trial is accessible at www.pactr.org. Written parental consent and assent for children above 7 years was obtained for each of the participants.

## Results

Of the 62 participants, 46 (74.0 %) were females. The median age of the respondents was 12 months (Interquartile range [IQR]: 8–36 months). The majority of the study participants were underweight with low weight for age. The median Z-score was 24 (Table 2).

**Table 2:** Socio demographic and pre-operative baseline characteristics of enrolled children

Characteristic	Category			*P* value
	All study	Drain	No drain	
	*N* = 62 (%)	*n* = 31 (%)	*n* = 31 (%)	
Age (months)	12 (8–36)	15 (8–51)	12 (6–36)	0.233
Gender*				
Female	46 (74.2)	20 (64.5)	26 (83.9)	0.08
Male	16 (25.8)	11 (35.5)	5 (16.1)	
Anthropometric measures				
Weight (Kg)	8 (6–15)	8 (5.7–16)	8 (6–12)	0.489
Height (Cm)	74.5 (65.5–101.5)	82 (69–105)	72.5 (61–88)	0.092
Underweight	24 (38.7)	12 (39.7)	12 (38.7)	1.0
Stunted	17 (27.4)	6 (19.4)	11 (35.5)	0.255
Wasted	23 (37.1)	12 (38.7)	11 (35.5)	1.0
Pulse rate (beats/min)	112 (102–130)	108 (99–120)	115 (103–131)	0.132
Blood pressure (mmHg)				
Systolic	90 (84–100)	90 (84–100)	90 (83–100)	0.688
Diastolic	45.5 (36–55)	42 (35–55)	47 (38–57)	0.330

*All data in median and interquartile ranges except if starred, where frequency and proportion (%) was used

Anthropometric status by World Health Organization cut-offs: Wasted is weight for length Z score (WLZ) < − 2; stunted is length for age Z score (LAZ) < − 2; and Underweight is weight for age Z score (WAZ) < − 2

For pleural space separation, in the no drain arm, 30 [96.7 %] participants compared to 31 [100.0 %] in the drain arm had pleural space separation of less than 30 millimeters (<30 mm) at 48 h (*P* = 1.000). Since no participant in the drain arm had pleural space separation of more than 30 mm, the measure of association by logistic regression could not be computed. The data were not sufficient to reject the null hypothesis that hypothesized no difference in significant postoperative pleural space accumulation between the drain and no-drain interventions.

Regarding surgical wound infections, in the no drain arm; none (0.0 %) of the patients had surgical wound infection compared to 5 (16.0 %) in the drain arm (*p* = 0.053). The measure of association between surgical wound infection and our intervention by logistic regression analysis could not be computed because of empty cell counts. Accordingly, the data were not sufficient to reject the null hypothesis that hypothesized no difference in surgical wound infection between the drain and no-drain interventions.

In relation to average oxygen saturation levels in liters per minute, our results indicated that 25 (80.7 %) of study participants in the no-drain arm in contrast to 18 (58.1 %) in the drain arm required oxygen supplementation levels of less than or equal to 1 l per minute (uOR: 0.33, 95 % CI: 0.11–1.04, *p* = 0.057).

More participants in the no-drain arm used less than 1 L/min of oxygen compared to those in the drain arm. However, after adjusting for age, sex, body-mass-index, length of thoracotomy incision, muscle sparing surgery and duration of surgery, this association was not statistically significant (aOR: 0.25, 95 % CI: 0.06–1.04, *p* = 0.057).

For the combined primary outcome (Table 3), data showed that; 27 (87.1 %) participants in the no-drain arm had significantly favorable outcomes (less adverse events) compared to 18 (58.1 %) in the drain arm. Further analysis indicated that participants in the no-drain arm were at reduced likelihood of having unfavorable outcome compared to the drain arm (uOR: 0.21, 95 % CI: 0.06–0.73, *p* = 0.015). Nonetheless, after adjusting for age, sex, body-mass-index, length of thoracotomy incision, muscle sparing surgery and duration of surgery, the “protective” effects were similar in both the drain and no-drain arm not statistically significantly associated (aOR: 0.07, 95 % CI: 0.00–2.50, *p* = 0.144).

**Table 3:** Logistic regression analysis of postoperative outcomes of surgery

Outcome	All study (*N* = 62)	Drain (*n* = 31)	No drain (*n* = 31)	Unadjusted			Adjusted for significant variables at unadjusted analysis		
				OR	95 % CI	^α^ *P* value	aOR	95 % CI	^α^ *P* value
Primary endpoints									
^a^Pleural separation (48 h)									
≤ 30 mm	61 (98.4)	31 (100)	30 (96.7)	–	–	1.000	–	–	–
> 30 mm	1 (1.6)	0 (0.0)	1 (3.3)	1.0					
^a^Surgical wound infection									
No	57 (91.9)	26 (83.8)	31 (100)	–	–	0.053	–	–	–
Yes	5 (8.1)	5 (16.2)	0 (0)						
Oxygen amount (L/min)									
≤ 1	43 (69.4)	18 (58.1)	25 (80.7)	0.33	0.11–1.04	0.059	0.25	0.06–1.04	0.057
> 1	19 (30.6)	13 (41.9)	6 (19.3)	1.0					
Combined primary endpoint									
Favorable	45 (72.5)	18 (58.1)	27 (87.1)	0.21	0.06–0.73	0.015	0.07	0.00–2.50	0.144
Unfavorable	17 (27.5)	13 (41.9)	4 (12.9)	1.0					
Secondary endpoint									
ICU stay (hours)									
≤ 24	56 (90.3)	27 (87.1)	29 (93.5)	0.47	0.08–2.75	0.399	–	–	–
> 24	6 (9.7)	4 (12.9)	2 (6.5)	1.0					

^a^Zero values in cells did not permit generation of Odds Ratios. Fisher’s exact test was employed for hypothesis testing, since expected values <5, by Cochran’s rules;(#)- used in computing the “revised” combined endpoint (unfavorable outcomes were pleural separation > 30 mm, surgical wound infection & oxygen consumption >1 L/min; (α) – *P*-value cut-off for one interim analysis (after 50 % recruitment) and one final analysis (after 100 % recruitment) is 0.029 using the Fixed Nominal Rule (see Table 2 above)

Concerning the length of stay in the intensive care unit (ICU), 29 (93.5 %) of the participants in the no-drain arm spent 24 h or less compared to 27 (87.1 %) in the drain arm. Univariable analysis indicated that participants in the no-drain arm had lower odds of staying for longer than 24 h in the ICU compared to participants in the drain arm, but this was not statistically significant (uOR = 0.47, 95 % CI: 0.08–2.75, *p* = 0.399).

## Discussion

Our study set to find out if children who did not receive a chest tube after PDA surgery were at put at higher risk of developing complications by assessing postoperative outcomes. The main outcomes evaluated in this study were retention of fluid and/or air in the pleural cavity, incidence of surgical site infections, oxygen saturation and ICU length of stay.

We found the pleural cavity remained free of fluid or air with or without the chest tube. In uncomplicated PDA ligation with minimal trauma and no damage to intrathoracic structures, the pleura effectively absorb the minimal inflammatory fluids. This therefore negates the use of routine chest drainage in uncomplicated PDA surgery [2]; a practice can be adopted in both low and middle-income countries. Furthermore our surgeons refused further randomization because they felt it was not justifiable to continue putting chest tubes for the children below 6 years whose drainage was always negligible.

The requirements for oxygen amounts in liters per minute between the drain and no-drain arms were similar. The minimum accepted oxygen saturation for every child post-PDA ligation was 92 %. In the study, we used saturations at or above 92 % to assess level of compromise of breathing as a result of the tube. Children were put at the lowest maintenance flows of less than or equal to 1 l per minute and adjusted upward if saturations dip below 92 %. Although oxygen requirements to achieve this saturation never differed significantly, nearly half of children with chest tubes inserted needed oxygen of more than 1 l per minute. This was more pronounced in the older children (84–144 months) in the drain arm because older children developed a splinting reflex where the chest muscles are tensed in response to pain [2]. This was because splinting reflex resulted into shallow ineffective breaths that made children require higher levels of oxygen in order to keep saturations above 92 %. However, it is important to note that there were only seven children in the group aged 84–144 months (7 to 12 years).

Our study indicated a higher proportion of children in the no drain arm did not accumulate significant pleural fluid. This was similar to a study on elective use of chest tubes in thoracotomies for congenital cardiovascular procedures [3]. One patient [3.3 %] in our no drain group developed a postoperative chylous effusion that had to be drained by tube thoracostomy. This is an expected complication in patients with abnormal anatomy of the lymphatic system as was with this child [12]. For this patient, a tube was inserted and his diet was restricted to low fat feeds. The fluid gradually reduced over 5 days and the tube was removed. The child recovered without any further complication.

Analysis of the data showed avoidance of the chest tube was associated with favorable outcomes. Essentially, our study indicated that the percentage of children with or without a chest tube drain that spent less than 24 h in the intensive care unit were comparable. This implied that the presence of the chest tube was not a determinant of prolonged ICU stay of more than 24 h because some participants stayed in the ICU after chest tube removal for the purpose of additional monitoring. Longer stay was mainly due to oxygen saturation being below 92 % or developing a febrile illness or if the surgeon required the child to stay for closer observation. This finding is important for low socio economic settings where ICU bed availability is limited and bed turnover needs to be high to cater for those who need the ICU.

Our RCT found surgical wound infections (sepsis) only occurred among participants with chest tube insertion. This was in 5 (16 %) participants in the chest tube arm and was seen as a yellow discharge at the exit site of the drain [13]. The thoracotomy and tube drain sites were dressed separately. The thoracotomy site was further reinforced with Dermabond® which strengthens the infection barrier. This was not so for the drain site. Wound opening for the thoracotomy site was done on the day of discharge (third postoperative day) or earlier if the dressing was heavily soiled with blood. This meant less handling of the site and less exposure to potential infectious organisms. No child in either group had purulent discharge at the thoracotomy site save for the normal inflammatory reaction (induration).

### Strengths and limitations

Our study was undermined by selection bias because we only got patients from the Uganda Heart Institute for treatment, which may not be representative. Our participants were also unblinded. We had few (seven) participants above 6 years of age so we cannot make strong conclusions for this age group. We did not culture the secretions from the drain site to ascertain infection or not. Furthermore, the surgeons refused any more randomizations so the study had to prematurely close. Nonetheless, our findings are from the largest randomized trial about PDA surgery conducted in a low-income setting and hence strengthen the evidence base for further research of decision making in practice.

## Conclusion

Our analysis suggested that avoiding routine use of chest drainage in children below 6 years was safe and did not predispose them to retaining symptomatic pleural fluid [14, 15].

Children who routinely received a chest drain were more likely to have higher oxygen needs and infection at the drainage site.

Chest tube insertion was not associated with prolonged stay in the Intensive Care Unit.

## Additional files

Additional file 1: Flow diagram for participants. (DOCX 36 kb) Additional file 2: PDA-POT-STUDY. (XLSX 32 kb)
