# Supplementary material for: Chest closure without drainage after open patent ductus arteriosus ligation in Ugandan children: A non blinded randomized controlled trial
Source: BMC Surg. 2016 Sep 29;16:69. doi: 10.1186/s12893-016-0182-x (PMC5041499; doi:10.1186/s12893-016-0182-x)
Supplement: Additional file 1: — Flow diagram for participants. (DOCX 36 kb) [file 12893_2016_182_MOESM1_ESM.docx]

FLOW DIAGRAM FOR PARTICIPANTS

Assessed for eligibility

(n=71)

# Enrollment

Excluded (n =9)

Not meeting inclusion criteria

(n =7)

Refused to participate

(n =2)

Randomized (n =62)

#

Allocated to intervention

DRAIN (n = 31)

Received allocated intervention (n = 31)

Allocated to intervention

NO DRAIN (n =31)

Received allocated intervention (n = 31)

**Follow up**

Analyzed (n = 31)

Excluded from analysis

(n = 0)

Analyzed (n = 31)

Excluded from analysis

(n = 0)

# Analysis

Lost to follow up

(n = 0)

Discontinued intervention (n = 0)

Lost to follow up

(n = 0)

Discontinued intervention (n = 1) developed a chylous effusion 48 hours after operation.

Allocation
